# Improving patient experience and safety at transitions of care through the Your Care Needs You (YCNY) intervention: a study protocol for a cluster randomised controlled feasibility trial

**DOI:** 10.1186/s40814-020-00655-5

**Published:** 2020-09-02

**Authors:** Ruth Baxter, Jenni Murray, Jane K. O’Hara, Catherine Hewitt, Gerry Richardson, Sarah Cockayne, Laura Sheard, Thomas Mills, Rebecca Lawton, on behalf of the PACT research team

**Affiliations:** 1 Yorkshire Quality and Safety Research Group, Bradford Institute for Health Research, Bradford, UK; 2 School of Healthcare, University of Leeds, Leeds, UK; 3 York Trials Unit, University of York, York, UK; 4 Centre for Health Economics, University of York, York, UK; 5 School of Psychology, University of Leeds, Leeds, UK

**Keywords:** Transitions of care, Discharge, Cluster randomised controlled trial, Hybrid interventions, Feasibility trial, Older people, Study protocol

## Abstract

**Background:**

Patients, particularly older people, often experience safety issues when transitioning from hospital to home. Although the evidence is currently equivocal as to how we can improve this transition of care, interventions that support patient involvement may be more effective. The ‘Your Care Needs You’ (YCNY) intervention supports patients to ‘know more’ and ‘do more’ whilst in hospital in order that they better understand their health condition and medications, maintain their daily activities, and can seek help at home if required. The intervention aims to reduce emergency hospital readmissions and improve safety and experience during the transition to home.

**Methods:**

As part of the Partners At Care Transitions (PACT) programme of research, a multi-centred cluster randomised controlled trial (cRCT) will be conducted to explore the feasibility of the YCNY intervention and trial methodology. Data will be used to refine the intervention and develop a protocol for a definitive cRCT.

Ten acute hospital wards (the clusters) from varying medical specialties including older peoples’ medicine, trauma and orthopaedics, cardiology, intermediate care, and stroke will be randomised to deliver YCNY or usual care on a 3:2 basis. Up to 200 patients aged 75 years and over and discharged to their own homes will be recruited to the study. Patients will complete follow-up questionnaires at 5-, 30-, and 90-days post-discharge and readmission data up to 90-days post-discharge will be extracted from their medical records.

Study outcomes will include measures of feasibility (e.g. screening, recruitment, and retention data) and processes required to collect routine data at a patient and ward level. In addition, interviews and observations involving up to 24 patients/carers and 28 staff will be conducted to qualitatively assess the acceptability, usefulness, and feasibility of the intervention and implementation package to patients and staff. A separate sub-study will be conducted to explore how accurately primary outcome data (30-day emergency hospital readmissions) can be gathered for the definitive cRCT.

**Discussion:**

This study will establish the feasibility of the YCNY intervention which aims to improve safety and experience during transitions of care. It will identify key methodological and implementation issues that need to be addressed prior to assessing the effectiveness of the YCNY intervention in a definitive cluster randomised controlled trial.

**Trial registration:**

UK Clinical Research Network Portfolio: 42191; ISTCRN: ISRCTN51154948. Registered 16/07/2019.

## Background

For older people and those with complex needs, the transitional period of moving from hospital to home poses various risks [1, 2]. As many as one in five patients experience an adverse event during this time, 62% of which could be prevented or ameliorated [3]. In recent years, emergency readmission rates have increased by 23% with around 30% of all readmissions estimated to be avoidable [4–6]. As older people are the highest users of the National Health Service (NHS) in the United Kingdom (UK), they represent an important target for support to improve transitions of care.

A recent meta-analysis of 92 randomised controlled trials (RCTs) of interventions to improve transitional care for older people observed a significant reduction in hospital readmissions at multiple time points up to 12 months post-discharge [7]. The existing interventions are all highly complex, adopting multiple and variable components, commencing, and ending at different time-points. As a consequence, deciphering which components are the active ingredients is challenging [8–10]. There is some suggestion however that interventions which seek to enhance patient capacity to reliably access and enact post-discharge care are effective [11, 12]. Supporting patient involvement aligns with the patient’s and carer’s position as the common denominator throughout the care pathway. Patient involvement in care during the hospital stay may be a key mechanism for enhancing patients’ capacity to ‘reach-in’ to the health care system enabling them to optimise their care [13]. The mechanism for doing this however has not been fully explored.

To address this knowledge gap, the Partners At Care Transitions (PACT) programme of research explores whether greater involvement of older patients and their families during the hospital stay can improve patient experience and safety at transitions of care. Through our earlier work, we have modelled transitional care to identify four key ‘functional aims’ or activities for which patients are responsible for (to varying degrees) once they are discharged home [14]. These include:

- Managing their health and wellbeing so that they understand what care they received in hospital and resume responsibility (as appropriate) for this at home;
- Managing their medications so that they have the knowledge and skills required to understand and take their medications correctly;
- Managing their daily activities to retain autonomy and minimise the effects of deconditioning;
- Escalating their care needs in an appropriate and timely manner.

Guided by our aim to explore patient involvement, we developed a ‘theory of change’ as to how transitions of care may be improved for older people [14]. We posit that supporting older people while they are in hospital to ‘practice being at home’ will better prepare them to manage these four functional activities at home. However, without support, patient involvement, particularly for older people, might be problematic and inconsistent. Patients themselves can fluctuate in how and when they want or are able to be involved, and busy staff may fail to recognise or engage with patient’s attempts to be involved [15].

Based on these principles, we co-designed an intervention, called ‘Your Care Needs You’ (YCNY), which aims to support older patients and carers to ‘know more’ and ‘do more’ whilst in hospital in relation to the identified four key functional aims. The resulting intervention aligns with emergent thinking about the development of complex interventions which focus on standardising interventions according to their functional aims rather than their form (i.e. specific components) [16]. Building on this, YCNY can also be regarded as a ‘hybrid’ intervention whereby certain components of an intervention are fixed, while others can vary according to the context within which they are implemented [17].

## Methods

### Study aims and objectives

This study aims to explore the feasibility of the YCNY intervention and trial methodology. In particular, the study will explore:

1. The acceptability, usefulness, and feasibility of intervention components and the implementation package to patients, carers, and staff;
2. The feasibility of methods to identify, recruit, and retain patients in the trial and to determine the best way to follow up patients in this population;
3. The required approaches for obtaining health economic data, ward level baseline data, and accurate routinely collected hospital emergency readmission data;
4. How intervention fidelity might be measured in the definitive cRCT.

Ultimately, findings from the study will be used to refine the intervention and inform the development of a protocol for the definitive cRCT in which the effectiveness and cost-effectiveness of YCNY will be assessed.

### Study design

A cluster randomised controlled feasibility trial will be conducted on ten wards (the clusters) within three acute NHS Trusts in the Yorkshire and Humber region of the UK. Cluster designs are used when there is a significant risk of contamination occurring if individual patients within a ward were randomised. Since this study includes a ward level component, it was not possible to use individual randomisation. Wards will be randomised to deliver either the YCNY intervention or usual care.

A minimum of 200 patients (20 per ward) will be recruited across intervention and control wards to complete questionnaires at baseline and then three time-points following discharge from hospital—5- to 14-, 30-, and 90-days post-discharge. Routine data regarding the patients’ index hospital admission and any subsequent readmissions (up to 90-days post-discharge) will be extracted from their medical record.

An embedded qualitative evaluation will assess the feasibility and acceptability of the intervention and implementation package. Up to 24 patients and their carers will be recruited from intervention wards to participate in patient-level observations of care and individual interviews. Up to 28 staff will be recruited to participate in interviews and ward-level observations.

In addition, a sub-study will be conducted alongside the feasibility trial to explore the most efficient, cost-effective, and accurate way of gathering primary outcome data (30-day emergency hospital readmissions) within the definitive cRCT. Approval has been sought from the Confidentiality Advisory Group (CAG) to access the medical records of 100 patients (10 per ward) without consent to understand the actual destinations of patients who are discharged to their ‘usual place of residence’.

**Fig. 1:**
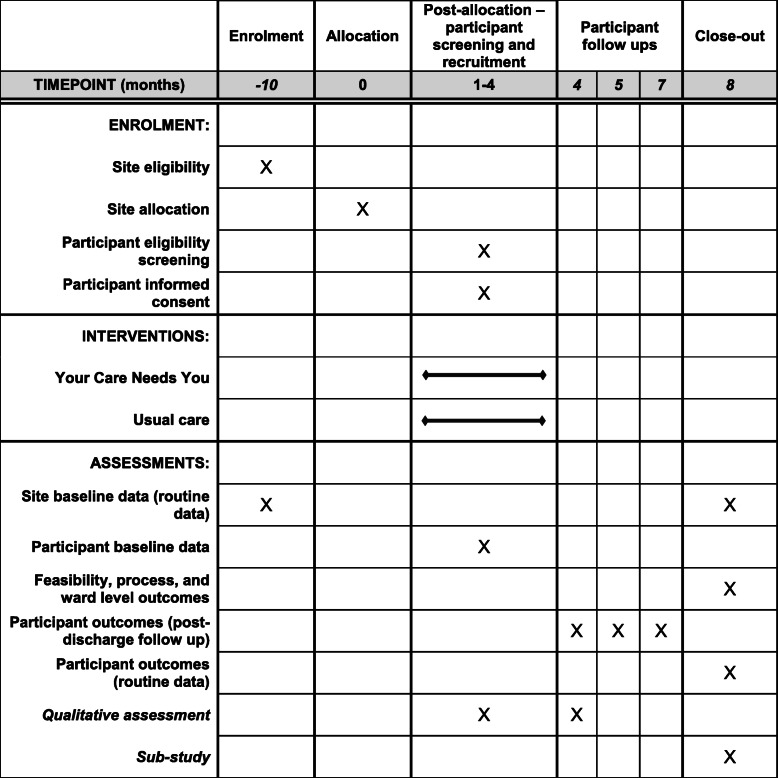
The schedule of enrolment, interventions, and assessments [as per Standard Protocol Items: Recommendations for Interventional Trials (SPIRIT)] [18]

Figure 1 details the schedule of study enrolment, interventions, and assessments. The SPIRIT checklist [18], which details the recommended items to include in a clinical trial protocol, is available in Additional file 1.

### Setting

The feasibility trial will be conducted across a range of specialties including older peoples’ medicine, trauma and orthopaedics, cardiology, intermediate care, and stroke. Eligible wards will be NHS funded, inpatient hospital wards where approximately 40% or more  of patients are routinely aged 75 years and over. Acute medical admission wards, wards without regular medical input, and/or those currently participating in a trial will be excluded. NHS Trust and ward level agreement will be gained for eligible wards to participate in the study.

### Randomisation

The randomisation will be carried out remotely by a statistician at the York Trials Unit (YTU), University of York who is not involved in the recruitment of wards or the enrolment of participants. Clusters (wards) will be randomised in an unequal allocation ratio (3:2) via minimisation using minimPy [19] (intervention *n* = 6; control *n* = 4). An unequal allocation ratio will ensure robust data for exploring the feasibility and acceptability of the intervention across a representative range of specialties. Naïve minimisation with a base probability 1.0 (i.e. deterministic minimisation) will be conducted using three key ward characteristics: ward specialty; percentage of patients aged 75 years and over; and NHS Trust. The wards will be informed of which arm they have been allocated by a member of the research team. The unequal allocation of wards prevents blinding of the treatment group.

### Patient population and sample size

Up to 10 wards (clusters) will be recruited to the study based on the need to have at least four clusters per arm to allow for basic statistical analysis [20]. As this is a feasibility trial, we did not undertake a formal sample size calculation. Based on previous experience of recruitment rates for older people in a recently completed trial [21] and to ensure that we will have sufficient numbers to explore the feasibility and test the intervention, we considered a minimum of 200 patients (20 per ward) would be sufficient. The assumptions underpinning this sample size will be assessed through the study. Eligible patients will be 75 years and over; anticipated to be discharged to their own home or that of a relative; staying for at least one night on a participating ward; able to read and understand English; and willing and able to give informed consent. If a patient lacks capacity, a personal consultee will advise on their wishes to participate. Patients will be considered ineligible if they live out of the area or plan to be transferred to another acute hospital, are admitted for psychiatric reasons (other than dementia/delirium), or are identified as being at the end of life.

### The Your Care Needs You (YCNY) intervention

#### Intervention components

The intervention comprises three fixed core components:

##### YCNY booklet

This patient-facing booklet is given to patients at the start of admission to a participating ward and is designed to be used throughout their stay and following discharge. Focusing on the four key functions, the booklet provides information as to why patients should ‘know more’ and ‘do more’ in hospital and gives suggestions as to how they could do this. The booklet contains ‘I would like to talk about’ pages which patients can use to indicate to staff that there is something they would like to discuss. There are spaces for patients, carers, or staff to make notes.

##### YCNY film

Where possible, a short educational film will be shown to patients when introducing the booklet. The film brings to life and seeks to underline our hypothesised links between retaining and supporting cognitive capability and physical capacity within the hospital, and better outcomes post-discharge. The web address of the film is publicised within the booklet so that carers/patients can also view it independently.

##### Care summary

At discharge, patients will receive a patient-friendly care summary, written in lay terminology, to help them understand why they have been in hospital, any changes to their medication, what to expect following discharge, and when and how to escalate care if needed. This letter will supplement rather than replace existing discharge letters which are sent to a patient’s General Practitioner (GP). Template letters will be provided for wards to flexibly implement according to their ward context.

In addition to these fixed core components, ward staff will be asked to consider how they currently support patient involvement with respect to the four key functions and what else they can do to enhance this. The actions that the teams decide to undertake will not be prescribed—they will be left to vary according to staff preferences, current activities/initiatives on the ward that already address the four functions, and their patient population.

#### Implementation

The implementation of YCNY will be supported in four ways.

*A facilitation meeting*, lasting 1–2 h, will be held with key staff members (e.g. ward manager, nurses, healthcare assistants, discharge coordinator, therapists, and doctors/consultants) to discuss delivery of the intervention within each wards’ specific context. Through the meeting, staff will (i) decide how to deliver the core intervention components; (ii) map what they currently do to meet the four functional aims; and (iii) think of additional tools or initiatives that they could use to meet these aims. A coach(es) will be identified whom the research team can work more closely with, and who can act as a point of contact for ward staff as needed.

*Deciding on staff roles—*To help the intervention fit into each ward’s specific context, the roles that staff groups will fulfil will vary. The roles required include introducing the YCNY booklet, showing the patient film, encouraging patient use of the booklet, and responding to patient questions. Roles will be discussed and allocated during the facilitation meeting.

*Staff training* will be delivered to multidisciplinary staff to ensure that they have knowledge of, understand the benefits of, and have the skills required to deliver the intervention. Training will be delivered through two principal routes: (i) fuller briefings about the intervention at extended multidisciplinary team meetings, staff handovers, or using ad hoc opportunities/drop-in sessions; and (ii) briefing and support for those involved in delivering specific intervention components (e.g. the care summary). Posters and handouts will help remind and support staff to interact with the booklet.

*A ‘share and learn’ session* will be held 2–3 weeks after the ward has started implementing the intervention. The session aims to identify and resolve any problems encountered in delivering the core intervention components, to reflect on how the booklet/film may have influenced patient behaviours (e.g. requesting to practice their medications) and to discuss how the team have and could respond to meet the functional aims of the intervention.

#### Control wards

Control wards will deliver usual care to their patients according to their Trust’s standard policies and procedures. Researchers will arrange short briefing sessions with the ward manager to inform the ward staff of the study activity and patient recruitment.

### Recruitment and consent

Recruitment will commence once ward teams have started to implement the intervention. A discretionary period of up to a month may be used to embed the intervention in the ward. Exact start dates for recruitment will vary by Trust and ward depending on when NHS Trust permissions are received and implementation is agreed at ward level. Recruitment is expected to take up to 4 months.

#### Screening and identification

Suitably qualified ward staff will screen and identify eligible patients. All patients aged 75 years and over will be screened for eligibility. Detailed screening logs will record numbers of eligible patients, key reasons for ineligibility, and recruitment/refusal rates. Screening data will be used to complete a CONSORT diagram for cluster trials [22].

#### Approach and consent

In addition to screening patients against the study’s inclusion and exclusion criteria, staff will be asked how and when eligible patients should be approached (e.g. whether they are well enough). Researchers will provide eligible patients with written information and a verbal explanation of the study (see Additional file 2 for an example). Patients will have an opportunity to ask questions and consider their participation. If patients wish to take part, a written (or witnessed) consent form will be completed. A letter will be sent to the patient’s GP to inform them of their participation.

#### Patients who lack capacity

A significant proportion of patients within this older population (75 years and over) are likely to lack the capacity to make decisions about their care. These patients are often more vulnerable to safety incidents and/or poorer experience during transitions of care [21]. To be as inclusive as possible, it is important that this particular patient population is included in the study. Capacity will be assessed during the screening process and the initial approach. If patients lack capacity, attempts will be made to identify and recruit an informal carer (e.g. family member or friend) who can act as a personal consultee for the patient. A written declaration will be gained from all participating consultees.

#### Withdrawal

The rights of patients and consultees to withdraw from the study will be respected. They can do so without giving a reason and without their care or treatment being affected. Data previously collected will be used in analyses (unless consent for this is withdrawn) and, where possible, a reason for withdrawal will be recorded. If patients are unexpectedly discharged from the hospital into 24-h care such as nursing/care homes (other than for a temporary period of rehabilitation), they will become ineligible and will be withdrawn.

### Outcomes

The main outcomes for this study are process-based, as the study aims to assess the feasibility of YCNY and the trial methodology, rather than the effectiveness of the intervention. Outcomes, therefore, include measures of feasibility namely: (i) screening, recruitment, and retention data; (ii) the processes required to collect accurate data from consented patients, routine medical records, and the ward (via information services); (iii) the approach required to collect health economic data; and (iv) qualitative data relating to the acceptability of YCNY and feasibility of implementing it. Patients will be asked to complete a number of validated measures which are outlined in the section below.

### Data collection

Data will be collected at the level of the patient and the ward through a mix of self-report and routinely collected data.

#### Patient baseline questionnaires

Following recruitment, researchers will support patients to complete a baseline questionnaire. This includes basic demographic information, contact details to conduct post-discharge follow-ups, the Barthel Index [23], EQ5D-5 L [24], and the Functional Comorbidity Index [25] (all validated measures are outlined in Table 1). Most data will be self-reported by patients or consultees but, where this is not possible, data will be gathered via staff or from patient medical records.

**Table 1:** Validated measures collected from patients at baseline and during post-discharge follow-ups

Measure	Description	Collected when
**Functional Co-morbidity Index (FCI)** [25]	18 self-reported comorbid conditions with a score of 0 to 18 with each item scoring 1. A higher FCI score indicates greater comorbidity and is associated with impairment in physical function 1 year later.	Baseline
**Barthel Index (BI)** [23]	10 items measuring a person’s daily functioning, particularly the activities of daily living and mobility. Total possible scores range from 0 to 20, with lower scores indicating increased disability.	Baseline
**EuroQol 5-Dimension Health Questionnaire (5 levels) (EQ5D-5 L) & Proxy EQ5D-5 L** [24]	The EQ5D-5 L and Proxy EQ5D-5 L measures quality of life comprising five dimensions: mobility, self-care, usual activities, pain/discomfort, and anxiety/depression. Each dimension is scored on a five-point ordinal scale: no problems, slight problems, moderate problems, severe problems, unable. Scores can be used to calculate quality-adjusted life-years (QALYs).	Baseline and post-discharge follow-ups
**Partners At Care Transitions Measure (PACT-M)** [26]	The PACT-M assesses patient perceptions of the quality and safety of transitions from hospital to home, relevant to a UK population. In total, eight items are scored on a five-point Likert scale: strongly disagree, disagree, neither agree nor disagree, agree, strongly agree with an additional option of ‘not applicable’. The PACT-M also measures the incidence (yes or no) of seven adverse events following discharge from the hospital.	Post-discharge follow-ups
**Care Transitions Measure 3 items (CTM-3)** [27]	The CTM-3 (derived from the 15-item CTM) is a patient-centred measure of the quality of care transitions. Three items are scored on a five-point Likert scale ranging from strongly agree to strongly disagree.	Post-discharge follow-ups
**Client Service Receipt Inventory (CSRI**) [28]	The CSRI will be used to assess patients’ use of health-related resources. Questions have been adapted to assess the health resources that are pertinent to care transitions from hospital to home for older people.	Post-discharge follow-ups

#### Patient post-discharge follow-up questionnaires

Following discharge from hospital, patients will be sent postal questionnaires at three time points—5-, 30-, and 90-days post-discharge. These time points represent a critical safety period (immediately post-discharge), are policy-relevant (30-days), and allow assessment of longer-term effects (90-days). To try and increase response rates, patients will receive a £5 voucher with the questionnaire at each time point [29]. Measures include the Partners At Care Transitions Measure (PACT-M) [26], Care Transitions Measure (3 items—CTM-3) [27], EuroQol 5-Dimension Health Questionnaire (5 levels) (EQ5D-5 L) and Proxy EQ5D-5 L [24], and the adapted Client Service Receipt Inventory (CSRI) [28] (see Table 1 for further details). Questions will also be asked to assess receipt and thereafter, the usefulness of the intervention.

**Fig. 2:**
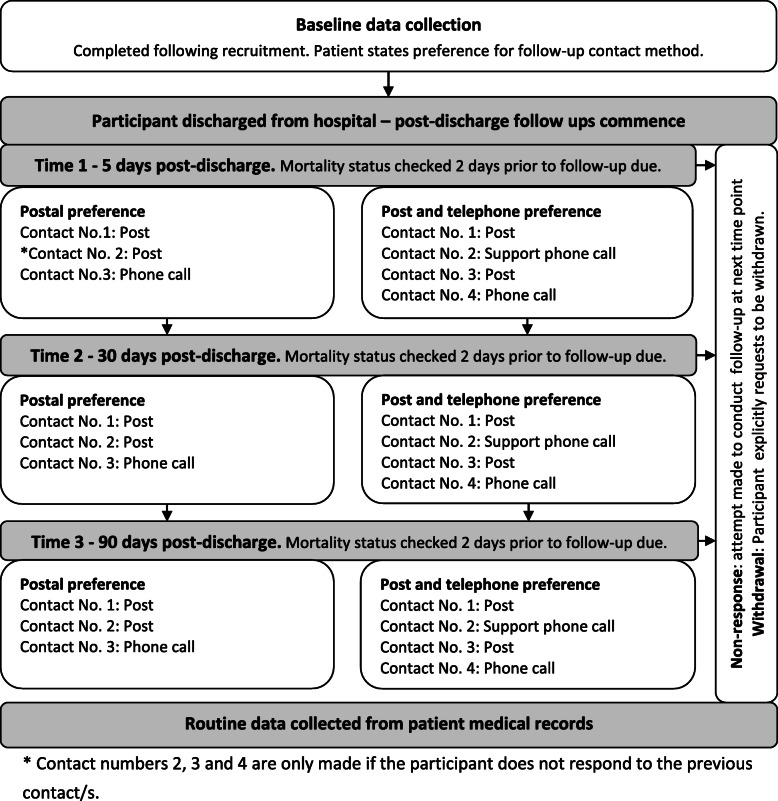
Process for collecting post-discharge follow-up data

Figure 2 outlines the process for collecting post-discharge follow-up data. Patients will choose to be followed up by post alone or to additionally receive telephone support. Those choosing the latter will be telephoned approximately 3 days after the first postal questionnaire is sent. If questionnaires are not returned, a postal reminder will be sent 10 days later and a final attempt to collect data will be made via telephone to all patients regardless of follow-up choice. We will attempt to collect data at each time point unless patients explicitly withdraw from the study. The mortality status of patients will be checked prior to each follow-up time point.

#### Patient-level routine data

In addition to the self-report data outlined above, routine data will be extracted from the consented patients’ medical records. Data includes:

- Recorded discharge date for the index admission—to ensure accurate discharge dates are recorded during the trial;
- Emergency hospital readmission dates up to 90-days post-discharge—to assess the feasibility of collecting our primary outcome measure in the definitive cRCT, and to calculate the length of stay for any readmissions;
- Ward moves that occurred prior to discharge during index admission—to assess intervention fidelity and contamination between wards.

Due to funding and progression of the PACT programme of work, the research team face tight timescales between finishing the feasibility trial and starting the definitive trial. As this study does not assess effectiveness, if the Trial Management Group decide that we already have enough data to assess the feasibility of progressing to a definitive trial, we may decide not to collect questionnaire and routine readmission data up to the full 90-days post-discharge for patients who are recruited later in the study.

#### Ward-level routine data

To explore what ward level baseline data are required for the definitive cRCT, the following non-identifiable data will be extracted for each participating ward over the most recent 12-month period:

- The total number of patients discharged from a participating ward and the total number of these patients who are readmitted to the hospital Trust (any ward) within a 30-day period (split by patients who are < 75 years and ≥ 75 years);
- Average length of stay for patients admitted to each participating ward (split by patients who are < 75 years and ≥ 75 years with monthly totals);

In addition, the total number of admissions to participating wards during the recruitment period (split by < 75 years and ≥ 75 years) will be used to identify how many patients aged 75 years and over were admitted to the ward but not screened.

### Statistical analysis

A full statistical analysis plan will be drafted before the completion of data collection. Quantitative analysis will focus on descriptive summaries for each treatment group including the number of wards and patients approached, randomly assigned, receiving intended treatment, and providing outcome data. A CONSORT diagram for cluster trials will document the flow of participants through the study. The percentage of participants readmitted will be determined. The degree of clustering using intracluster correlation coefficients (with 95% confidence intervals) may also be quantified, acknowledging that this may be unreliable due to the small sample size [30].

Participant withdrawals, reasons for withdrawal, and non-responses at each data collection point will be summarised and compared by trial arm. Recruitment and attrition rates (ward and patient) across the two arms will be compared, along with outcomes data at follow-up. All baseline and outcome data will be summarised descriptively by trial arm using means and standard deviations for continuous variables and counts and percentages for categorical data. Participants will be summarised by their randomised ward irrespective of whether they received the actual allocation or not. PACT-M data will also be analysed to define cut-offs for a high versus low-quality transition. There are no planned interim analyses.

Qualitative and quantitative data will be used to assess the following criteria when deciding whether to progress to a definitive cRCT:

- 2–8 eligible wards exist within participating trusts (depending on size) and narrative indications of staff interest
- Average of 4 patients per ward per month recruited
- Attrition less than 10% for the primary outcome
- Staff, patients, and carers positively evaluate the intervention (or modifications can be made to address any concerns).

Fulfilment of these progression criteria will be discussed and agreed with our Trial and Programme Management Groups, our independent Trial Steering Committee, and the funders (the National Institute for Health Research).

### Embedded qualitative assessment of feasibility

Qualitative methods will be embedded within the feasibility study to explore the acceptability, usefulness, and feasibility of the intervention, and explore how fidelity can be assessed.

#### Sample

On intervention wards, purposive and opportunity sampling will be used to recruit a nested sample of 20–24 patients (3–4 patients per ward). Patients will consent to patient-level observations and interviews in addition to the quantitative post-discharge follow-ups. Carers (e.g. spouse or child) who are involved in a patient’s care will also be recruited to participate. Patients will receive up to £30 as a thank you for their participation.

Multidisciplinary staff involved in the facilitation meetings and implementing the intervention will be purposively sampled to participate in interviews and/or ward-level observations. In addition, one staff member on each control ward will be recruited at the end of the study to participate in an interview (*n* ≤ 28).

#### Data collection methods

##### Interviews

Semi-structured interviews will be conducted with patients, carers, and staff to explore acceptability, usability, usefulness, and feasibility of the intervention components (both fixed and flexible) and to explore how implementation worked within the ward context. Suggestions about optimising the intervention and implementation package will be gathered. Patient interviews lasting 30–60 min will take place just after discharge and will be conducted either at the patient’s home or over the telephone. If patients continue to use the intervention at home, a second interview will be conducted at 30-days post-discharge. Staff interviews will last 15–30 min. Interviews with staff on control wards will explore any activities that are similar to those that would meet the functional aims of the intervention (e.g. End PJ Paralysis Initiatives) and thus appear to ‘dilute’ the effect of the intervention, or serve as evidence of contamination. Interviews will be audio-recorded where possible, otherwise, detailed field notes will be taken.

##### Observations

Patient- and ward-level observations will explore how patients, carers, and staff interact with the intervention during the patient stay and at discharge. Patient-level observations will include the introduction of the booklet, occasions where staff and patients interact as part of routine care (e.g. ward and medicine rounds), and at discharge. Ward-level observations are likely to include the facilitation session and support required to embed the intervention, staff training, staff roles in using the booklet, and ward level activities (e.g. daily cares, ward rounds, medication rounds, meetings, briefings, and handovers). Observations will be captured through field notes and ‘contact summary forms’ will be completed for each ‘contact’ (e.g. a discrete piece of observation, or something less bounded such as a day spent observing ward activity).

##### Documentary analysis

All participants who consent to the quantitative arm of the feasibility trial will be asked at the 30-day post-discharge follow-up if they received a YCNY booklet in hospital and, if so, whether they would be willing to return it to the research team. Any returned booklets will be analysed to explore how they have been used.

#### Qualitative data analysis

Qualitative data will be analysed using a ‘pen portrait’ method [31]. Pen portraits are typically used to synthesise data across sources. Data related to each ward will be drawn together to describe how the intervention was implemented, how staff and patients engaged with the intervention, and what the experience was for patients following discharge. Pen portraits will subsequently form the unit of analysis and be subjected to a thematic analysis [32], before being synthesised, with coding specifically pertaining to issues of acceptability and feasibility.

### Sub-study to assess discharge destinations

The target patient population for this programme of work is older people who transition from hospital back into their own home (rather than 24-h care, e.g. a nursing or care home). To test the effectiveness of the intervention in the definitive cRCT, primary outcome data (30-day emergency readmissions) are required from 7000 patients which we had planned to gather at a ward level via routinely collected administrative data. However, our previous work has highlighted that patients are coded as being discharged to their ‘usual place of residence’ which does not distinguish those who go from and to their own homes (our target population) and those who go from and to 24-h care/nursing homes. Not understanding the size of this coding problem represents a major threat to the internal validity of the definitive trial. As such, a separate sub-study will explore the actual discharge destinations of patients on participating wards who are aged 75 years or over and are coded as being discharged to their ‘usual place of residence’.

Confidentiality Advisory Group permissions have been gained to access patient medical records without consent. Trusts will identify a consecutive sample of 10 patients from each participating ward who are aged 75 years and over and have been coded as discharged to their ‘usual place of residence’. Their medical records will be accessed to identify their actual discharge address and this will be categorised as being either: their own or a relative’s home; a nursing or care home; intermediate care; or other. The research team will receive anonymised, aggregated ward-level data detailing the total number of patients discharged to each category.

### Data management, monitoring, and safety reporting

Patient data will be recorded on case report forms (CRFs). Participants will be assigned a unique identification number and all data will be completely anonymised for purposes of analysis and reporting. Electronic data and wet ink copies of the CRFs will be stored securely at YTU. Data will be monitored for quality and completeness by YTU. Missing data, except that from post-discharge follow-ups, will be chased until they are received or confirmed as not available. Most data will be collected directly from participants and so cannot be subject to data verification. All qualitative data and consent forms will be held securely by the Yorkshire Quality and Safety Research Group (YQSR) at the Bradford Institute for Health Research.

The trial is overseen by the Trial Management Group (TMG) comprising of the chief investigator, key co-applicants, and the operational members of YQSR and YTU. An independent Trial Steering Committee (TSC) and Data Monitoring Committee (DMC) comprising academic, NHS England, clinical, and a patient representative meet annually. Further details are available on request.

In this patient population, acute illness, deterioration of health, and readmissions are likely and so will not be reported during this study. The death of participants is also expected. All expected and unrelated deaths will be reported to the TMG and reported annually to the REC and TSC/DMC. Any unexpected and related serious adverse outcomes will be reviewed by the chief investigator and reported to the sponsor and ethics committee.

### Trial organisation and administration

The feasibility study is being conducted as part of a five and a half year Programme Grant for Applied Health Research (RP-PG-1214-20,017) funded by the National Institute for Health Research. The trial is sponsored by Bradford Teaching Hospitals NHS Foundation Trust and is coordinated by YQSR at the Bradford Institute for Health Research, and YTU at the University of York.

Approvals were gained from the Wales Rec 7 Research Ethics Committee, Confidentiality Advisory Group, and the Health Research Authority prior to starting the study (REC reference [19]/WA/0162, CAG reference [19]/CAG/0105). Local NHS capability and capacity approvals were granted by all participating NHS Trusts. Any amendments to the protocol will be submitted for the required regulatory approval. The study is registered on the UK Clinical Research Network Study Portfolio (42191) and the ISTCRN (ISRCTN51154948).

### Patient and public involvement

The PACT programme grant has an active patient panel who have been involved in co-designing the YCNY intervention, and have advised and supported the development of study procedures and documents. Panel members will be involved in the analysis, dissemination of results, intervention iteration, and protocol development for the definitive cRCT.

### Dissemination

Findings will contribute to the on-going progression of the PACT programme of work. They will be disseminated widely to a broad audience including academics, clinicians, healthcare managers, policymakers, and patients and the public and participants within the study. The findings will be written up for publication in peer-reviewed journals and will be presented at national and international conferences, workshops, and learning events.

## Discussion

Transitions from hospital to home can be risky, particularly for older people who often have complex health and/or social care needs [3]. Although the evidence is equivocal, there is some suggestion that interventions that support patient involvement may improve transitional care outcomes [10, 12]. The PACT programme of research, therefore, asks whether supported involvement of older patients and their families in their care improves patient experience and safety at transitions of care. Through our earlier work [14, 33, 34], we have designed an intervention which supports patients to ‘know more’ and ‘do more’ during their hospital stay so that they can manage their care at home post-discharge. The present study assesses the feasibility of delivering this intervention and the trial methodology in order to refine the intervention and protocol for a definitive cRCT trial. As with any complex intervention various challenges outlined below are anticipated throughout the study.

### Recruitment

The study will assess the feasibility of implementing the intervention and recruiting patients on different types of the ward (i.e. different acute specialties). It may be that some specialties (e.g. stroke care) are less suited due to their specified patient pathways, provision of extensive post-discharge rehabilitation, and/or low patient turnover. Further to this, patient movement between intervention, control, and other hospital wards prior to discharge will be assessed in order to explore issues of contamination and fidelity.

### Post-discharge follow-ups

Challenges are also anticipated when conducting post-discharge follow-ups. First, the study team will be reliant on participating Trusts regularly tracking patient discharges to trigger the processes for post-discharge follow-ups. Patients who are discharged to their home address may do so via a different address. Some of this variation will be observed by tracking discharge destinations (e.g. to intermediate care beds) but some of it may not (e.g. if a patient temporarily stays at a relative’s home). The proportion of unreturned questionnaires due to non-receipt by the patient or another reason may remain unknown. Second, over recent years, the lengths of hospital admissions have reduced [35] and so patients are increasingly going home with ongoing care needs such as wound care management or medication monitoring. Collecting data during the initial post-discharge period when patients may still be unwell and/or particularly vulnerable to hospital readmissions [36] may impact our CRF return rates. Third, retention and attrition will need to be monitored as follow-ups are being conducted up to 90-days post-discharge. Up to 15% of patients are likely to be readmitted within 30 days [37] which is likely to impact patients’ ability to respond to questionnaires accurately and within the necessary time frames. In addition, attrition is expected as people request to withdraw or become lost to follow-up. Death within this older patient population is also expected during the follow-up period.

### Implementation

A key aim of this study is to assess the feasibility of the intervention and implementation package. The qualitative evaluation will be conducted to monitor the use of the intervention by staff, patients, and carers in order to identify barriers and enablers. These data will be used to refine the YNCY intervention and implementation package used in the definitive trial. As hybrid interventions are relatively novel within health services research [17], the qualitative data will also be of particular importance in guiding the research team on how they can support flexible implementation within the confines of a cRCT study design.

### Trial status

This article refers to protocol version 3 dated 19/11/2019. Recruitment began on 15/11/2019 with completion expected by the end of March 2020. Post-discharge data collection is due to finish in June 2020.

This feasibility trial will support the identification of key methodological issues that will be faced when implementing and assessing the effectiveness of the YCNY intervention. The results of the study will be used to develop a protocol for the definitive cRCT which is due to commence in the summer of 2020.

## Supplementary information

**Additional file 1.** Improving patient experience and safety at transitions of care through the Partners at Care Transitions (PACT) intervention: a study protocol for a cluster randomised controlled feasibility trial.

**Additional file 2.** Partners At Care Transitions (PACT): improving patient experience and safety at transitions of care.

## Data Availability

Requests to access the PACT data should be made to the corresponding author and will be considered on a case-by-case basis by the Chief Investigator and Trial Management Group. All data requests for quantitative data will be managed in accordance with YTU, University of York, processes and procedures.

## Abbreviations

CAG: Confidentiality Advisory Group
CONSORT: Consolidated Standards for Reporting Trials
cRCT: Cluster randomised controlled trial
CRFs: Case report forms
CSRI: Client Service Receipt Inventory
CTM: Care Transitions Measure
DMC: Data Monitoring Committee
GP: General practitioner
NHS: National Health Service
NIHR: National Institute for Health Research
PACT: Partners At Care Transitions
RCT: Randomised controlled trial
QALYs: Quality-adjusted life-years
TMG: Trial Management Group
TSC: Trial Steering Committee
UK: United Kingdom
YCNY: Your Care Needs You
YQSR: Yorkshire Quality and Safety Research group
YTU: York Trials Unit
