# Supplementary material for: Improving patient experience and safety at transitions of care through the Your Care Needs You (YCNY) intervention: a study protocol for a cluster randomised controlled feasibility trial
Source: Pilot Feasibility Stud. 2020 Sep 2;6:123. doi: 10.1186/s40814-020-00655-5 (PMC7466784; doi:10.1186/s40814-020-00655-5)
Supplement: Supplementary file 2 — Additional file 2. Partners At Care Transitions (PACT): improving patient experience and safety at transitions of care. [file 40814_2020_655_MOESM2_ESM.docx]

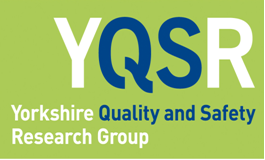

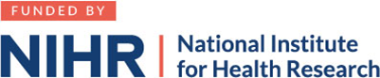

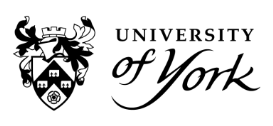


Trust logo

Partners At Care Transitions (PACT): Improving Patient Experience and Safety at Transitions of Care


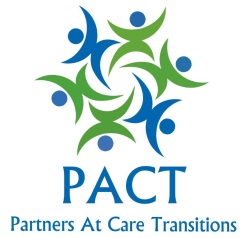


Information Leaflet for Patients (a)

Version 3, 8/8/19

Our research team is trying out ways to help older people get home from hospital safely and help them to stay there. This ward is taking part in a research study called PACT (Partners At Care Transitions). This leaflet tells you about the study, what we would like you to do, and how we would manage the information. Please read this carefully and ask us if anything is not clear or if you would like more information.

What is the purpose of the study?

Research has shown that people often go home from hospital without fully understanding what has happened to them. As a result people can find the first few weeks at home difficult and sometimes they go back into hospital when it could have been avoided. To improve the safety and experience of older people returning home from hospital we are trying different ways to help them get home and stay at home. On this ward, people are being given a ‘Your Care Needs You’ booklet and care summary to help them know and do more while they are in hospital. You may also be shown a video.

Why have I been chosen?

You have been invited to take part in this study because you are aged 75 or older and have been admitted to a hospital ward that is taking part in this study.

What will I be asked to do if I take part?

We will ask you some questions about yourself and we will contact you three times: at around one week, one month and three months after you get home from hospital. We will ask how you are managing at home and about your health and wellbeing after your hospital stay. We can ask you these questions over the telephone or we can send a questionnaire to you in the post. You can choose. If you choose a telephone questionnaire, we will ring you at a time that is convenient to you. If you choose the posted questionnaire, you will need to post it back to us and we will provide a pre-paid envelope. We are interested in what you think and feel so there are no right or wrong answers.

We would also like your permission to access your medical record so that we can collect basic information about you, e.g. your diagnoses and whether you have had to come back into hospital. We will also let your GP know that you are taking part in the study.

Do I have to take part?

No, you do not have to take part. Your care will not be affected by your decision in any way. If you decide to take part and later change your mind, you can leave the study at any time by contacting Ruth Baxter or Jenni Murray (details below). If you stop taking part, the information already collected from you will be included in the final study. You can ask for it to be removed–your care will not be affected.

What are the benefits of taking part?

There are no direct benefits to taking part in the study. However, it may help improve future services and support for people like you. As a small token of our appreciation for your continued support of the study you will receive a gift voucher with each questionnaire.

What are the risks of taking part?

We do not think that there are any disadvantages/risks to completing the questionnaires. You do not have to answer any questions you do not want to, and you can leave the study at any time by contacting Ruth Baxter or Jenni Murray (details below). We will not share your private information with third parties at any time.

What will happen to my information?

All of the information we collect from you will be securely stored at the Yorkshire Quality and Safety Research Group at the Bradford Royal Infirmary and/or University of York. All information will remain confidential. However, if we have concerns that you, or someone else, is at risk of harm then we may break confidentiality and tell the relevant healthcare services.

We will publish our findings in academic journals and present at conferences. You will not be identified in any report/publication. If you would like to know the findings of our study, we will need to keep your name, address, and date of birth until we have the results so that we can post something to you. We would keep your details securely and destroy them when we have sent you the findings. This would be no later than December 2020.

Who is organising and funding the research?

This study is organised and run by the Yorkshire Quality and Safety Research Group at Bradford Teaching Hospitals NHS Foundation Trust. It is funded by the National Institute for Health Research.

How will the study comply with the new General Data Protection Regulations (GDPR)?

Bradford Teaching Hospitals NHS Foundation Trust (BTHFT) is the sponsor for this study based in England. We will be using your information to undertake this study and will act as the data controller for this study. This means that we are responsible for looking after your information and using it properly. BTHFT will keep identifiable information about you for 6-12 months after the study has ended.

Your rights to access change or move your information are limited, as we need to manage your information in specific ways in order for the research to be reliable and accurate. If you withdraw from the study, we will keep the information about you that we have already obtained. To safeguard your rights, we will use the minimum personally-identifiable information possible. You can find out more about how we use your information at <https://www.bradfordhospitals.nhs.uk/our-trust/our-policies-and-procedures/>

BTHFT will collect information from you for this research study in accordance with our instructions. BTHFT will make sure that relevant information about the study is recorded for your care, and to oversee the quality of the study. Individuals from BTHFT and regulatory organisations may look at your medical and research records to check the accuracy of the research study. The only people in BTHFT who will have access to information that identifies you will be people who need to contact you to as part of taking part in the research, or audit the data collection process.

Who has reviewed the study?

This study has been reviewed and approved by Wales REC 7 Ethics Committee (reference number 19/WA/0162, date approved 24/09/2019).

What if I need more information or there is a problem?

If you have any questions or concerns you can speak with your researcher. You can also contact: Ruth Baxter (01274 383421 or [Ruth.baxter@bthft.nhs.uk](mailto:Ruth.baxter@bthft.nhs.uk)); or the Programme Manager Dr Jenni Murray (01274 383667 or [Jenni.Murray@bthft.nhs.uk](mailto:Jenni.Murray@bthft.nhs.uk))

**Thank you for reading this information sheet.**

**Partners at Care Transitions (PACT):**

**Improving Patient Experience and Safety at Transition of Care**

| **CONSENT FORM (a) for patients** | | Please  initial box |
| --- | --- | --- |
| 1. | I confirm that I have read and understood the Information Leaflet for Patients (a) (Version 3, 8/8/19) for the above study. I have had the opportunity to ask questions. |  |
| 2. | I understand that my participation is voluntary and that I am free to withdraw at any time without giving a reason and without my medical care or legal rights being affected. |  |
| 3. | I understand that relevant sections of my medical notes and data collected during the study may be looked at by individuals from the Yorkshire Quality and Safety Research Group, regulatory authorities or from the NHS Trust, where it is relevant to my taking part in this research. I give permission for these individuals to have access to my records. |  |
| 4. | I understand that my GP and/or community care team will be informed that I am taking part in the study. |  |
| 5. | I understand that any data I provide may appear in research publications. I will not be identifiable in any reports or publications from this study. |  |
| 6. | I agree if I withdraw from the study, the information already collected from me will contribute to the study, but I can ask for it to be removed. |  |
| 7. | I understand that a copy of this Consent Form will be stored at the Yorkshire Quality and Safety Research Group and/or University of York. |  |
| 8. | I understand that the information held and maintained by the Yorkshire Quality and Safety Research Group may be used to help contact me or acquire information about my health status. |  |
| 9. | **I agree to take part in this study.** |  |

**PARTICIPANT:**

NAME (capitals) DATE SIGNATURE

--------------------------------- ------------------------- -------------------------------

**RESEARCHER:**

NAME (capitals) DATE SIGNATURE

--------------------------------- -------------------------- -------------------------------
